# Study on Ergonomic Design of Artificial Intelligence Lower Limb Assist Brace for the Elderly

**DOI:** 10.1155/2022/3304513

**Published:** 2022-08-05

**Authors:** Zhen Yu

**Affiliations:** Science and Art Integration Research Center, China Academy of Art, Hangzhou 310009, China

## Abstract

The ergonomic design study of artificial intelligence lower limb-assisted brace for the elderly is a new design standard of lower limb-assisted brace for the elderly with mobility problems. Based on human factors engineering, this study tested and analyzed the advantages and disadvantages of human lower limb motion mechanics, human gait motion law, and existing lower limb assisted brace design cases at home and abroad and concluded that the common external assisted method is less man-machine efficient than the internal assisted method. Therefore, a new brace joint rotation curvature, component parameters, and other key information were designed based on the structure of the medial assistance method. With the help of the engineering and scientific analysis methods in human factors engineering, the designed machines and systems are made more adaptable to the physiological and psychological characteristics of human beings. This study explores the interaction between humans and machines and the rationality of their mutual integration, which can effectively avoid repetitive strain injuries and other muscle diseases over time for users in the process of assistance and achieve efficiency, health, and safety. Subsequently, Rhino software was used for digital modeling, physical prototyping, experimental testing, and analysis of the design solution and continuous optimization of the design. At the same time, the perceptual engineering design method was utilized to meet the humanized aesthetic design requirements. The prototype of the design study was finally completed, which is more in line with the evaluation criteria of “human-machine-environment system” than the existing market design in terms of functional rationality, human-machine performance, and human experience. This demonstrates the validity of the design method and is an important reference for the design standard of the lower limb support for the elderly.

## 1. Introduction

Since 2014, China's population aged 60 years and above has been growing, and the aging process in China has accelerated, and the Chinese population has now entered the elderly type. At the end of 2021, China's population aged 60 and above will be 267.36 million, accounting for 18.9%, an increase of 3.29 million compared to 2020. Among the 267.36 million people aged 60 and above in China in 2021, the population aged 65 and above will be 20.56 million, an increase of 9.92 million or 14.2% from 2020. The proportion of population aged 60 and above and 65 and above in China will be 18.7% and 13.5%, respectively, in 2020; the proportion of population aged 60 and above and 65 and above in China will increase by 0.2 and 0.7 percentage points, respectively, in 2021 compared with 2020, and the degree of aging will further deepen [1]. As the aging of the elderly population deepens and the cost of rehabilitation treatment for the elderly increases, the operational load of social nature care institutions such as homes for the elderly, hospitals, and rehabilitation institutions and the cost of care for their families will be greatly increased, and the pressure and resistance to social development will be further expanded.

Lower extremity exoskeleton has been proven to be efficient to provide highly repeatable and accurate rehabilitation exercise, but most existing exoskeletons' gait trajectories will not vary with the users [2]. In order to cope with this global social phenomenon and problem, many countries in the world have already developed lower limb assistance braces for the elderly. Along with the continuous research and development of related products, the technology of lower limb assistance brace has been continuously improved. However, the current product technology on the market tends to be homogeneous and expensive, and its design ideas are relatively solidified, and the suitability design standard has not been effectively established and not effectively combined with today's new industrial technology capabilities, module integration capabilities, and humanized use demand scenarios. This has led to low patient coverage and the inability to effectively exploit the functional advantages of this type of product. Ultimately, the research field cannot be developed sustainably, resulting in a delay in the development of the research field. Under the same level of assistance, the lower limb assistance brace with more ergonomic structure and design aesthetics can improve the experience of the elderly; through a more reasonable structural design to reduce product falsification, it can make the elderly have a more suitable assistance product. This study digs deep into user pain points and industry needs and fully verifies the rationality of product design ideas from multiple perspectives, including domestic and international industry status research, human factors engineering methods, modeling and structure design, suitability design, and computer simulation testing, and proposes new solutions. The design research direction can effectively supplement and enrich the research field of lower limb assistance brace at home and abroad, fill the relevant industry gaps, promote the development of artificial intelligence lower limb assistance brace industry, and become a new development trend and design standard of lower limb assistance brace in the future.

### 1.1. Status of Foreign Research on Artificial Intelligence Lower Limb Brace

**Figure 1:**
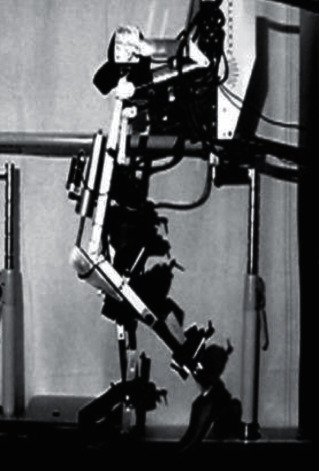
Lokomat lower limb gait training brace.

Lokomat is a lower limb gait training brace system used to enhance the mobility of the elderly. It consists of a human gravity support, a gait corrector, and a running table, and the parameters of Lokomat are adjusted to simulate the different physiological gait trajectories of different elderly people with mobility problems and to drive the elderly's lower limbs for walking training in Figure 1 [3].

The ALEXI lower limb assistance brace from the United States uses a nonlinear filter to assist the elderly to walk according to a preset trajectory [4].

**Figure 2:**
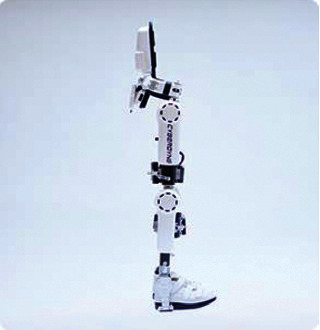
HAL lower extremity assist brace.

The HAL lower extremity assist brace from Cyberdyne, Japan, provides gait training and lower extremity walking assistance for the elderly in Figure 2 [5]. The structure of HAL is a lateral traction brace that is strapped to the leg of the elderly and uses biosensors to monitor the bioelectrical signals on the leg muscles to enhance the strength and stability of walking.

**Figure 3:**
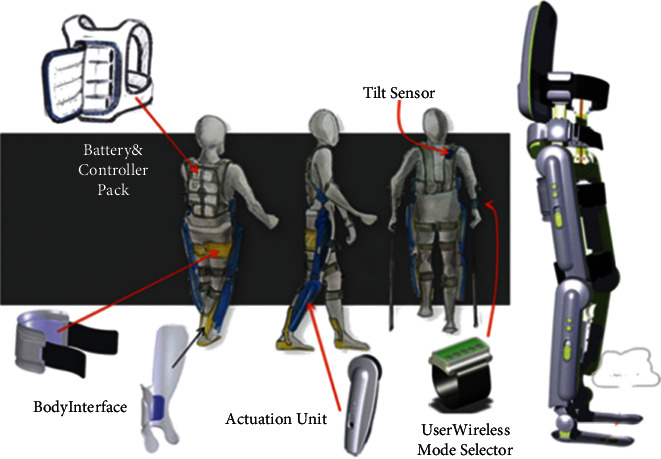
ReWalk lower extremity assist brace.

The ReWalk lower extremity assist brace is also a lateral traction assist brace to assist the elderly in walking (see Figure 3) [6].

Ekso Bionics is a lower limb assistance brace developed for military, medical, and other fields. Ekso has built-in high-precision sensors, anthropomorphic skeletal joints, microdrive motors, a powerful central processor, and drive software system.

**Figure 4:**
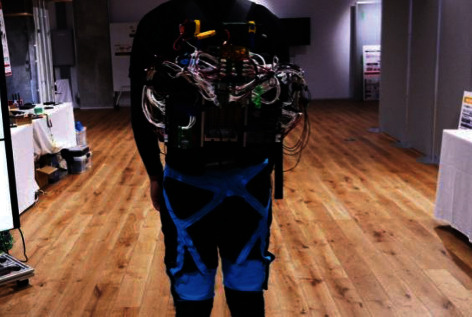
Inflatable lower limb assistance brace developed by Panasonic.

The inflatable lower limb support brace developed by Panasonic uses soft mechanical principles to support a variety of lower limb muscle movements and compared to the traditional lower limb support brace. The quality is also lighter and easier to wear, and there is still a certain distance from the mass production of products (see Figure 4) [7].

### 1.2. Status of Domestic Research on Artificial Intelligence Lower Limb Power-Assisted Brace

**Figure 5:**
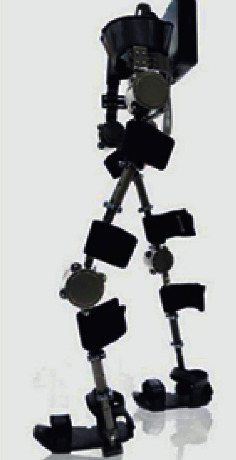
Ailegs Aide lower limb power-assisted brace.

Shanghai Jiaotong University fuses surface EMG signals and interactive forces into a lower limb-assisted brace [8]. Beijing DAI's Ailegs lower extremity assist brace is made of titanium alloy and adopts the form of assisted structure with external traction, strapped to the leg of the elderly, which can be adjusted in size to fit different body types of the elderly and can bear up to 100 kg of weight. Shanghai Fourier X1 lower limb assistance brace integrates mechanics, human biosensor, electromechanical drive integration, gait analysis, and other multidisciplinary science and technology to help the elderly achieve basic assistance functions such as sitting, standing, walking, and going up and down stairs (see Figure 5) [9]. The auto-LEE lower limb assist brace from the Shenzhen Institute of Advanced Technology of the Chinese Academy of Sciences can drive each component independently and work together to assist the elderly to walk without the assistance of other devices compared to the traditional assist brace [10].

## 2. Overview of Human Factors Engineering

Human factors engineering, also known as “Human Factors Engineering”, is an interdisciplinary discipline involving human factors engineering, perceptual engineering, engineering, psychology, physiology, anthropometry, anatomy, environmental science, system science, management, safety science, labor science, and other disciplines. It has been widely applied in many fields.

Human factors engineering takes human as the core factor and emphasizes the factors that prioritize human needs in engineering and work management. By systematically studying basic human data such as information on human abilities, behaviors, limitations and characteristics, and parameters of various behaviors and by systematically applying these data elements to the design and manufacture of products, operating procedures, and the environment, in which they are used, we study the interaction between humans and machines and solve the problem of the efficiency of collaboration between humans and machines. With the help of robots and intelligent systems, artificial intelligence technology can improve problems such as physical and human constraints; for example, during human-robot collaboration, robots can use human biomechanical models and habitual motion trajectories to adjust work patterns and consider a variety of human factors engineering check indicators, including joint torque, body posture, mechanical stress, lower limb maneuverability, and muscle fatigue levels. The interaction between the “human-machine-environment system” is optimized so that its operation is compatible with the physiological composition and psychological needs of human beings. Therefore, human factors engineering research is often accompanied by the application of perceptual engineering. By quantifying the various perceptual factors of human beings in an engineering way, the relationship between each perceptual quantity and the product design is matched and finally transformed into the physical design elements of the product.

Human factors engineering enables human beings to act efficiently, safely, conveniently, healthily, and comfortably under different conditions in activities such as living, working, and leisure by analyzing and changing the interrelationships between human beings and human behavior and the products, equipment, facilities, procedures, systems, and their associated environments used.

## 3. Design Method of Lower Limb Assistance Brace for the Elderly

### 3.1. Gait Detection and Data Analysis of the Lower Limbs of the Elderly

Gait detection and analysis are crucial in the ergonomic study of the lower extremity of the elderly. The lower limb gait of the elderly includes walking, running, going up and down stairs, and other periodic movements, in which the legs alternate and move forward to drive the body forward, among which the most frequently used movement is walking, in which the legs and limbs need to be highly coordinated with each other. In order to design a fully applicable auxiliary brace for the lower limbs of the elderly it is necessary to master the design of various parameters at different levels of gait movements of different elderly people through in-depth research on the gait movement rules of the elderly. The synchronized study of basic human data of the elderly, together with various parameters such as information related to the ability, limitation, and characteristics of gait movement of the elderly, can provide the engineering data support for the design of the lower limb assistance brace for the elderly in a reasonable and suitable range.

The whole-body muscles need to be involved in the process of gait movement of the elderly, coordinating with the hip, knee, and ankle joint rotation and flexion, tilt and rotation of the trunk, and the lateral, longitudinal, forward, and backward movement of the human body, which is a complex movement of the elderly body. The three main joints of hip, knee, and ankle are the basic joints in the gait movement of the lower limbs of the elderly, while other lower limb joints play more or less the role of regulation.

In the detection of gait motion of the elderly, the macroscopic view is that the torso is the main body of the whole movement, and the two lower limbs are in the subordinate position; microscopic view is that the thighs belong to the main body, and the lower legs are in the subordinate position, in decreasing order, and they are connected by the joints that play the role of mutual connection and support, so the range of activities of each independent joint is calibrated according to the need of human factors engineering, and the body movement pattern of the elderly is obtained after a sampling test. In the walking process of the elderly, a gait motion cycle starts from the landing of one foot to the end of the landing of the foot again. The process includes a number of movement phases such as foot landing, single foot support, single foot off the ground, and single foot swing. There is basically no difference in the proportion of each phase among older adults of different genders, ages, and heights. Firstly, the group randomly selected a certain range of sample subjects among different types of elderly people, then continuously observed the samples, objectively recorded walking duration, movement direction, movement speed, displacement path, and other movement data, categorized and analyzed the data, and finally mapped the data in the corresponding three-dimensional spatial range, which reduced the instability brought by individual differences and could improve the accuracy of the lower limb brace research work.

**Figure 6:**
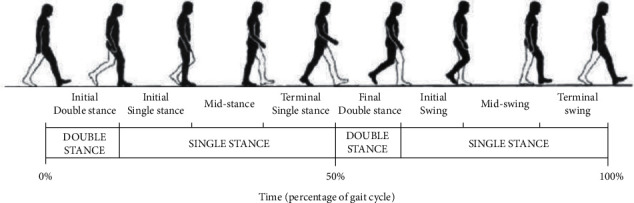
Gait cycle diagram for the elderly.

The gait study of the elderly utilizes foot sensors, and the subject team collects the movement trajectory and time of the elderly walking to quantitatively analyze and assess the kinematic and kinetic parameters of gait cycle and mechanics, mainly including the parameters of stride length, stride width, stride frequency, and joint operation angle (see Figure 6) [11]. For example, the normal value of stride length is 1500∼1600 mm. The normal value of step width is 50∼100 mm. The rotation angle of the foot joint is 0∼7°. And this is used to construct the gait motion model.

The regularity, parameters, and periodicity of normal human gait motion are relatively stable although there are very slight individual differences. However, as the joints of the lower extremities of the elderly age or develop disease, the gait motion characteristics will change significantly. According to the principle of human factors engineering, this requires the design process to customize different lower limb brace assistance structures and modes according to the aging degree of different elderly people's lower limbs and therefore requires the lower limb assistance brace to have the function of independent adjustment of joints.

### 3.2. Human Factors Engineering Research Method Intervention

The design and development of the artificial intelligence lower limb brace follow the ergonomic design principle. Thanks to the new industrial technology capability and module integration capability, the lower limb brace can be more humanized, i.e., the integration and humanization of the artificial intelligence lower limb brace body when it is used with people is improved.

Using the principle of ergonomics, this paper studies the changes of motion posture, the position of support points and the changes of comfort of people with the same height, size, and weight in various scenes, and finally establishes the parameter model of human dynamic support.The experimental test data and results show that the most reasonable relative position between human comfort and integrated support structure components, anthropomorphic joints, hydraulic devices, power motors, and transmission systems can reduce power transmission losses, improve the ability of auxiliary actions, and achieve the most comfortable auxiliary use.

**Figure 7:**
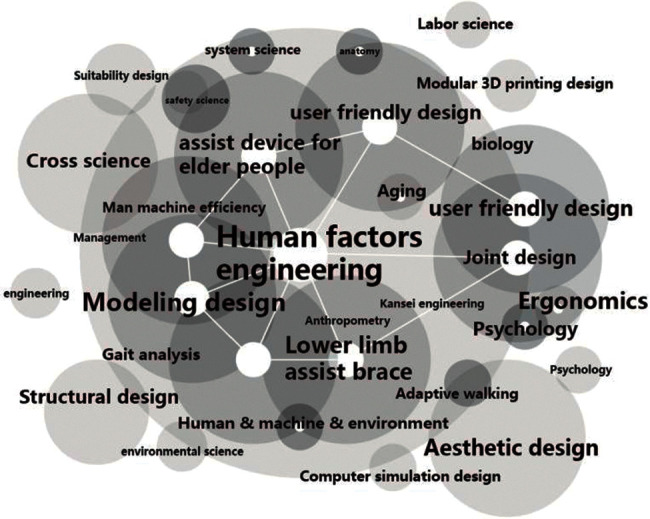
Project main technology diagram.

As most of the existing artificial intelligence skeleton is based on the lateral skeleton leg support, which leads to too many restrictions on the range of activities and poor human-computer interaction experience, therefore, the artificial intelligence-assisted lower limb development project can use experience, human-computer interaction, temperature and mechanical structure, control model, power module, new materials, and other aspects to carry out innovative research and design research. The main technologies are shown in Figure 7.

### 3.3. Modeling and Structural Design

**Figure 8:**
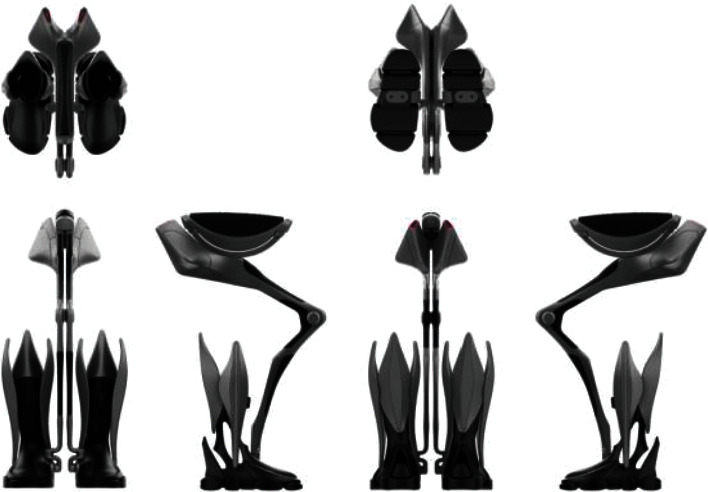
Product shape design diagram.

The modeling design of the elderly artificial intelligence lower limb assistance brace needs to meet the functional needs, suitability needs, and potential humanized sensibility needs of consumers (see Figure 8). The research direction is to adopt the medial assistance method. Compared with the traditional external traction assistance method, it can be changed into the form of support assistance. This kind of assistance, such as the auxiliary unicycle, can effectively share the weight of the elderly when walking, standing, squatting, climbing stairs, and other actions, so that users have a more relaxed and comfortable experience. It can prevent the traditional structure of long-term use that will lead to the body of the brace and the human body in contact with the skin compression, and blood is not smooth.

This structure should not only have advantages in the external product function design but also have excellent sensory visual effect in the product appearance. Compared with the traditional booster structure, the main components are hidden in the inner measurement of the legs, which can use the legs to cover the main bracket structure and have a better performance in terms of aesthetics.

In terms of suitability requirements, the structure is designed to be lightweight by bionic design, concerning animal bones and biological structures under the premise of fully considering the rationality of its structure, and the weight is reduced by hollowing out some parts, reducing the three circumferences, and changing the combined structure, while the cost of materials can be reduced, making it less burdensome for the elderly to purchase and use the product under the premise of satisfying the lower limb support function and strength requirements.

Moreover, in terms of humanized sensual needs, according to the principle of sensual engineering design, compared with the traditional traction assistance method, which uses a machine to control people and tug them forward, this design method is more like a tool that can ride, which can transform the tool controlling people into the psychological implication of people controlling the tool.

The research of artificial intelligence lower limb assistance brace for the elderly needs to make comprehensive reference to the advantages and disadvantages of the existing international and domestic cases, as well as the real use experience and feedback of the elderly, to exclude the unreasonable and even hazardous parts of the existing design for the health of the elderly and to make preliminary screening. In addition, we should fully consider the living habits of the elderly and the habits of using the lower limb brace and design the structural and functional solutions according to the gait motion parameters of the elderly in order to meet the basic lower limb assistance function of the elderly and also meet other physiological and psychological needs of the elderly.

**Figure 9:**
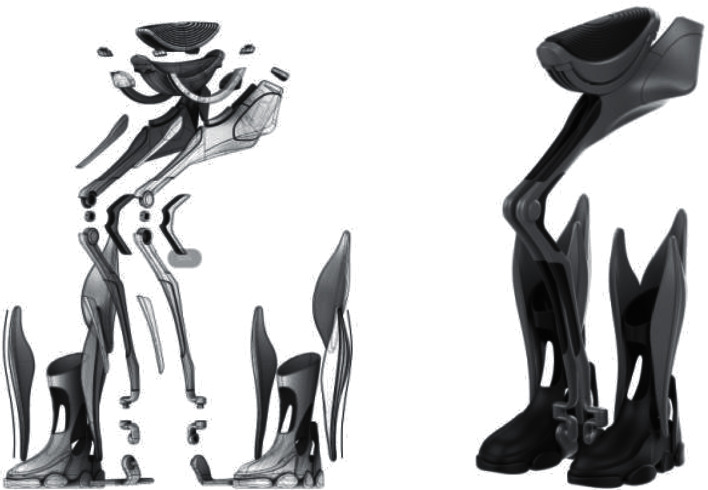
Structural components design diagram.

The main structure and joints are realized by 3D printing technology, and the main mechanical structure consists of hip seat support, hip joint, knee joint, ankle joint, exoskeleton module, foot module, and other parts (see Figure 9).

Adopting the design of a new structure, compared with the traditional traction type wearable lower limb power-assisted brace and suspension type wearable lower limb power-assisted brace, using a new technology, highly integrated, humanized design method, designed a new brace joint rotation curvature, component parameters, and other key information, using Rhino software for three-dimensional modeling, physical prototyping, experimental testing analysis, and continuous optimization of the design plan. The design of the brace is designed to solve the problems of the user's movement being restricted due to the complex and large design of the body, the uncomfortable wearing due to the lack of ergonomic design, and the need for the user to overcome the psychological acceptance of wearing the brace.

The lower extremity brace for the elderly needs to be reasonably configured based on the degree of freedom and joint drive range of the brace, taking into account the degree of matching with the human lower extremity, joint drive, and system complexity. The driving power of hip joint and driving distance of knee joint are in great demand, while the ankle joint is the motion end of the lower limb brace, and the position changes frequently and randomly. Therefore, the hip and knee joint degrees of freedom in the sagittal plane are assigned to the booster drive, i.e., the active drive is used for the hip and knee surface/extension; the ankle joint is configured as passive degrees of freedom, i.e., the ankle joint is a flexible passive joint, which maximizes the advantage of “man in the ring” and brings into play the ability of man in maintaining balance and reduces the difficulty of mechanism design and control. It reduces the difficulty of mechanism design and control. The joint range of motion of the human lower limb horizontal walking and the joint range of the booster are shown in Table 1.

## 4. Suitability Design

From the perspective of suitability design, the modular design of the artificial intelligence lower extremity brace for the elderly can reduce the upgrade cost by replacing only the modules for functional upgrade. The products on the market are limited by the limitations of earlier technical standards and design ideas, and the integrated structure is used to ensure the functional integrity and reliability of the whole. The modular design can effectively avoid a series of problems brought about by the integrated design, such as larger volume and weight, a single way to assist action, maintenance difficulties, repurchase of new equipment, environmental protection, and poor economy. The use of modular design can make the lower extremity assistance bracket with the potential for subsequent functional upgrades, in order to achieve richer and more efficient functions without having to buy a new product can be replaced on the original equipment or pretend to new functional modules. At the same time, the modular design, if the equipment is damaged, only the corresponding damaged modules or parts need to be replaced, without the overall disassembly of the body for maintenance, low maintenance difficulty, low zero to whole ratio, high success rate of maintenance, and short maintenance time, and even the elderly can replace the repair themselves according to the manufacturer's instructions.

### 4.1. Lightweight, Integrated

At present, a large number of mechanical structures, power systems and power transmission systems are used in the market to drive the support to help the body. One of the two drawbacks is that the brace itself has a large number of structural components and uses metal or alloy materials to ensure the structural strength, and the overall mass is huge. Its power system needs to share a lot of energy to support its own weight, in order to use the remaining power reserves to help the body, resulting in low efficiency and poor range. Second, the structural components are complex and nested in layers. This limits the angle, amplitude, and distance of the support, and because the body is too large, the range of motion and angle of human limbs is also limited.

There is a large amount of material redundancy in the middle part of the traditional brace structure, which can be hollowed out to reduce weight and optimize the structure design of the lower limb brace components. At the same time, on this basis, a large numbers of aluminum alloy, titanium alloy, carbon fiber, graphene, and other lightweight composite materials are used in the key parts to further reduce the overall mass.

Combined with ergonomic principles, the combination of new materials is improved to simplify the structural components and achieve a highly integrated structural design. Compared with the existing research direction, while ensuring the structural reliability of the exoskeleton, it can greatly reduce the self-weight of the lower limb power support, enhance the payload capacity, reduce the burden of wearing, and enhance the flexibility and comfort of wearing.

### 4.2. Modular 3D Fabric Printing Design

At present, the lower limb support for the elderly at home and abroad is mainly to meet the basic functions, ignoring the humanization and use experience, as well as the lack of ergonomic design, which leads to the possibility that the force area and position of human exoskeleton worn by different users are unreasonable. The exoskeleton body will lead to wear and tear of the skin in contact with the human body.

The body and foot moving parts are designed with modular customization, and 3D printing technology is applied to realize the customization of muscle body, which can achieve a higher conformity to the body auxiliary parts from human factors engineering, and customize the product according to the user's bone size data, so that the man-machine model can cope with the different needs of the elderly with different gender, age, body size, and degree of mobility.

In addition, the 3D smart knitting machine can realize the digital molding of various colors, patterns, thicknesses, and other design factors to achieve the goal of personalized customization of the bracket component units. It maximizes the physiological and psychological needs of elderly users. From the perspective of humanized sensual engineering design, the elderly will not just be satisfied with the ordinary assistance mode of traditional products. Help different needs of the elderly to enhance limb mobility and expand the range of action and action time, which can expand the intelligent scenario and has the same important significance for the development of artificial intelligence lower limb assistance brace and human society.

## 5. Computer Simulation Test

**Figure 10:**
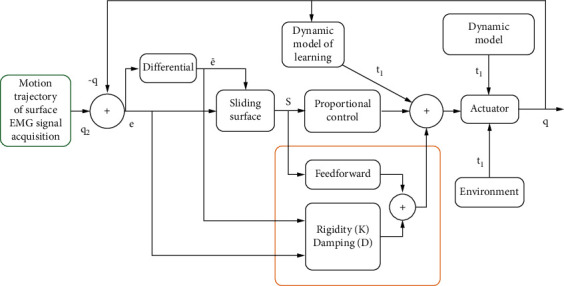
Control algorithm flow chart.

The artificial intelligence lower limb assisted brace for the elderly uses an adaptive control method similar to the human-machine learning observer to allow the machine to learn the kinetic model feedforward that the system should have. This control method allows the lower limb assisted brace to overcome the damping of the motion process to guide the patient to complete the required motor movements and gradually reduce the force according to the patient's completion, until the lower limb assisted brace is completely free of force and completely through the patient's own muscle force to complete the desired motor movements, to achieve the “suitable walking state “function (see Figure 10). At the same time, this algorithm can also identify the robot's motion model and perform feedforward compensation during the motion.

The control software is the core of the whole set of lower limb assistance brace system for the elderly, which provides three modes for users to choose, walking mode, up and down stairs mode, and ProStep (automatically sensing the user's body movement to trigger each step); the elderly can choose according to their own situation and assistance progress. At the same time, the product can walk through the intelligent bracket to achieve real-time data monitoring, and count and upload relevant data to assist medical personnel in analysis and use.

**Figure 11:**
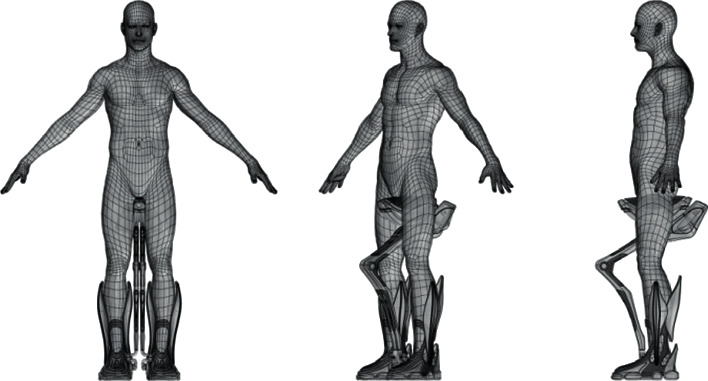
Human-machine simulation experiment diagram.

With the help of computer 3D software and related simulation software, the product is adjusted between shape and structure. Combined with the “suitable walking state” function, the computer software simulates the changes in the movement posture and force state of people of different body types and people of the same body type with different strengths under various scenarios of movement conditions. Test and record the position, area size, and morphological changes of the support contact surface that the testers feel comfortable with under different assistance strengths, and build a model of human dynamic support force parameters according to these data parameters. A variety of exoskeleton structural components of various sizes and forms were prototyped with reference to the force parameter models and tested. Based on the experimental test data and results, the most versatile structural parts that meet the comfort and integration of the human body and the exoskeleton body can be derived from the experimental test data and results, so as to arrive at the optimal design direction and design scheme. The method of computer simulation can improve the efficiency and quality of design demonstration, shorten the research period, and reduce the cost of research and development. The human-machine simulation experiments using computer software are shown in Figure 11.

## 6. Conclusion

Based on human factors engineering, this study analyzed and researched the existing artificial intelligence brace lower limb brace at home and abroad and proposed a new design method for the structure of the brace and the ergonomic design standard of the lower limb brace. It makes it possible to meet the humanized design principles of the elderly on the basis of meeting the lower limb gait movement assistance of the elderly.

The comprehensive design methods of ergonomic design research, user analysis, engineering analysis, behavior study, scenario analysis, functional design, structural design, and modeling design of the lower limb assisted brace for the elderly artificial intelligence brace are demonstrated. The rationality was verified by computer simulation and experimental prototype testing, which provided an important theoretical basis and prototype modeling reference for a new design standard in the field of lower limb assistance brace for the elderly.

Through this study, the importance and feasibility of human factors engineering in the research field of lower limb assistance braces for the elderly are reflected. The future research direction of the artificial intelligence-assisted brace for the elderly lies in the cross-collaboration of multiple disciplines under the continuous accumulation and transformation of design thinking and high technology. It can be said with certainty that the humanization, comfort, applicability, and convenience design of the lower limb assistance brace based on the ergonomics of the elderly are an inevitable trend. The elderly will therefore have more ergonomic assistance products and be more willing to walk with the help of braces, which can greatly improve the quality of life, work efficiency, and happiness of the elderly group.

## Figures and Tables

**Table 1:** Joint range of motion and range of joint involvement of the assisted brace.

Joint	Degree of freedom	Human walking joint range of motion	Range of joint design of the brace
Knee	Flexion/extension	−125°∼15°	
	Flexion/extension	−130°∼ 0°/0°	−120°∼ 0°
Size of each file	Max. 5 mb	−20°∼ 0°/0°∼ 45°	−20°∼ 20°

## Data Availability

The data used to support the findings of this study are available from the corresponding author upon request.
